# Supplementary material for: Exploring psychiatrists’ perspectives on supporting parents with mental health Challenges: A mixed-methods study
Source: PLoS One. 2026 Feb 17;21(2):e0342923. doi: 10.1371/journal.pone.0342923 (PMC12912598; doi:10.1371/journal.pone.0342923)
Supplement: Attachment — Submitted filename: Response to reviewer comments-3.docx [file pone.0342923.s002.docx]

We are grateful to the Academic Editor for the thoughtful and valuable comments. Please find our detailed response below.

**First Editor’s comment:**

1. We note that there were multiple versions of Manuscript in your submission's file inventory. Please double check your submission file inventory retaining the most recent version(s) for editorial review and let us know if any files appear to be outdated or absent.

**Response to Editor’s comment:**

We removed all manuscript files in the portal, retaining only a version with tracked changes.

**Second Editor’s comment:**

2. We note that you have indicated that there are restrictions to data sharing for this study. For studies involving human research participant data or other sensitive data, we encourage authors to share de-identified or anonymized data. However, when data cannot be publicly shared for ethical reasons, we allow authors to make their data sets available upon request. For information on unacceptable data access restrictions, please see <http://journals.plos.org/plosone/s/data-availability#loc-unacceptable-data-access-restrictions>.

If there are ethical or legal restrictions on sharing a de-identified data set, please explain them in detail (e.g., data contain potentially identifying or sensitive patient information, data are owned by a third-party organization, etc.) and who has imposed them (e.g., a Research Ethics Committee or Institutional Review Board, etc.). Please also provide contact information for a data access committee, ethics committee, or other institutional body to which data requests may be sent.

Please update your Data Availability statement in the submission form accordingly."

**Response to Editor’s comment:**

Thank you for your email and for your comments regarding the data availability statement for our manuscript. After carefully reviewing our ethical approval documents, we unfortunately do not have the required participant consent to make the full dataset publicly available, even in de-identified form. Public data sharing was not included in our original information and consent form, and the Research Ethics Committees of the Université du Québec en Outaouais and of the Centre intégré universitaire de santé et de services sociaux (CIUSSS) de la Capitale-Nationale have therefore restricted us from making these data publicly accessible.

In line with PLOS’s guidance that data requests should be directed to a non-author institutional point of contact, we propose the university’s Ethics Committee as a neutral, non-author institutional contact for controlled access to the data, even though data management is not part of its formal mandate. Data requests may be addressed to:

Université du Québec en Outaouais Ethics Committee
Email: [comite.ethique@uqo.ca](mailto:comite.ethique@uqo.ca)

Accordingly, we propose the following Data Availability statement:

“Data cannot be shared publicly because participants did not provide consent for open data sharing, the dataset contains potentially identifying and sensitive information, and the Research Ethics Committee has restricted public access to the dataset. Data are available from the Université du Québec en Outaouais Ethics Committee (contact via [comite.ethique@uqo.ca](mailto:comite.ethique@uqo.ca)) for qualified researchers who meet the criteria for access to confidential data.”

Thank you for your understanding. Please let us know if further clarification or adjustments are needed.

Best regards,

Geneviève Piché

Geneviève Piché, Ph.D., ps.éd. | PROFESSEURE TITULAIRE

Département de psychoéducation et de psychologie - Campus de Saint-Jérôme

Université du Québec en Outaouais

[genevieve.piche@uqo.ca](mailto:genevieve.piche@uqo.ca)
